# Prevalence of Dyslipidemia in Urban and Rural India: The ICMR–INDIAB Study

**DOI:** 10.1371/journal.pone.0096808

**Published:** 2014-05-09

**Authors:** Shashank R. Joshi, Ranjit Mohan Anjana, Mohan Deepa, Rajendra Pradeepa, Anil Bhansali, Vinay K. Dhandania, Prashant P. Joshi, Ranjit Unnikrishnan, Elangovan Nirmal, Radhakrishnan Subashini, Sri Venkata Madhu, Paturi Vishnupriya Rao, Ashok Kumar Das, Tanvir Kaur, Deepak Kumar Shukla, Viswanathan Mohan, for the ICMR– INDIAB Collaborative Study Group

**Affiliations:** 1 Lilavati Hospital, Mumbai, India; 2 Madras Diabetes Research Foundation & Dr. Mohan's Diabetes Specialities Centre, WHO Collaborating Centre for Noncommunicable Diseases Prevention and Control & IDF Centre of Education, Chennai, India; 3 Postgraduate Institute of Medical Education and Research, Chandigarh, India; 4 Diabetes Care Center, Ranchi, India; 5 Indira Gandhi Government Medical College, Nagpur, India; 6 University College of Medical Sciences and GTB Hospital, Delhi, India; 7 Nizams Institute of Medical Sciences, Hyderabad, India; 8 Department of Medicine, Jawaharlal Institute of Post-Graduate Medical Education and Research, Puducherry, India; 9 Indian Council of Medical Research, New Delhi, India; Mayo Clinic, United States of America

## Abstract

**Aim:**

To study the pattern and prevalence of dyslipidemia in a large representative sample of four selected regions in India.

**Methods:**

Phase I of the Indian Council of Medical Research–India Diabetes (ICMR-INDIAB) study was conducted in a representative population of three states of India [Tamil Nadu, Maharashtra and Jharkhand] and one Union Territory [Chandigarh], and covered a population of 213 million people using stratified multistage sampling design to recruit individuals ≥20 years of age. All the study subjects (n = 16,607) underwent anthropometric measurements and oral glucose tolerance tests were done using capillary blood (except in self-reported diabetes). In addition, in every 5th subject (n = 2042), a fasting venous sample was collected and assayed for lipids. Dyslipidemia was diagnosed using National Cholesterol Education Programme (NCEP) guidelines.

**Results:**

Of the subjects studied, 13.9% had hypercholesterolemia, 29.5% had hypertriglyceridemia, 72.3% had low HDL-C, 11.8% had high LDL-C levels and 79% had abnormalities in one of the lipid parameters. Regional disparity exists with the highest rates of hypercholesterolemia observed in Tamilnadu (18.3%), highest rates of hypertriglyceridemia in Chandigarh (38.6%), highest rates of low HDL-C in Jharkhand (76.8%) and highest rates of high LDL-C in Tamilnadu (15.8%). Except for low HDL-C and in the state of Maharashtra, in all other states, urban residents had the highest prevalence of lipid abnormalities compared to rural residents. Low HDL-C was the most common lipid abnormality (72.3%) in all the four regions studied; in 44.9% of subjects, it was present as an isolated abnormality. Common significant risk factors for dyslipidemia included obesity, diabetes, and dysglycemia.

**Conclusion:**

The prevalence of dyslipidemia is very high in India, which calls for urgent lifestyle intervention strategies to prevent and manage this important cardiovascular risk factor.

## Introduction

Cardiovascular disease (CVD) is the leading cause of death worldwide, and mortality due to CVD is higher in low- and middle-income countries [1], [2]. In India, there has been an alarming increase in the prevalence of CVD over the past two decades so much so that accounts for 24% of all deaths among adults aged 25–69 years [3]. Asian Indians have been found to develop CVD at a younger age than other populations [4]. The likely causes for the increase in the CVD rates include lifestyle changes associated with urbanization and the epidemiologic and nutritional transitions that accompany economic development [5]. Dyslipidemia has been closely linked to the pathophysiology of CVD and is a key independent modifiable risk factor for cardiovascular disease [6], [7]. While Asian Indians are known to have a unique pattern of dyslipidemia with lower HDL cholesterol, increased triglyceride levels and higher proportion of small dense LDL cholesterol, there have been no large scale representative studies on dyslipidemia to assess the magnitude of the problem in India. The estimation of the prevalence of dyslipidemia will ensure proper planning of health care resources for both primary and secondary prevention of CVDs. This article will report on the lipid patterns and prevalence of lipid abnormalities of the Indian population studied in Phase I of the Indian Council of Medical Research India Diabetes Study (ICMR-INDIAB study), involving three states and one union territory (UT), representing the north, south, east and west of the country.

## Methods

The methodology of the ICMR-INDIAB study has been published separately [8], [9]. Briefly, this is a cross-sectional survey involving adults aged 20 years and above (age range: 20–90 years). The study plans to survey all the 28 states in India, the two Union Territories (UT) of Chandigarh and Puducherry and the National Capital Territory (NCT) of Delhi in a phased manner. Phase I of the ICMR-INDIAB study was conducted from November 2008 to April 2010, and included three states randomly selected to represent the south (Tamilnadu), west (Maharashtra), and east (Jharkhand) of India and one union territory (UT) representing northern India (Chandigarh). These four states have a population of 213 million, which is roughly 1/8 of India's total population of 1.2 billion people. In INDIAB-NE, the 8 north eastern states namely Sikkim, Assam, Meghalaya, Tripura, Mizoram, Manipur, Nagaland and Arunachal Pradesh are being sampled and in phase II, 5 other states from the rest of India are currently in progress. This paper presents results of Phase I of the study.

In all phases, a stratified multistage sampling design was followed [similar to the National Family Health Survey-3 (NFHS-3)]. The Primary Sampling Units (PSUs) were villages in rural areas and Census Enumeration Blocks (CEBs) in urban areas. A three-level stratification process was done using geography, population size and socio-economic status. Using a precision of 20% (80% power) and allowing for a non-response rate of 20%, the sample size was calculated to be 4,000 per state/UT (2,800 rural and 1,200 urban) [9]. Thus, the sample size for the entire study once completed would be 1,24,000 (28 states, 2 UTs and 1 NCT) and the sample size for the Phase I of the study was estimated to be 16,000 individuals.

In both urban and rural areas, one individual was chosen from the selected household following the World Health Organization (WHO) KISH method. A total of 16,607 individuals (5,112 urban and 11,495 rural) were selected from 363 PSUs (188 urban and 175 rural), of whom, 14,277 individuals responded (response rate 86%).

### Ethics statement

Approval of the Institutional Ethics committee, of the Madras Diabetes Research Foundation (MDRF), was obtained prior to study commencement and written informed consent was obtained from all study subjects in the local language.

### Data collection

In all study subjects, an interviewer-administered questionnaire was used to obtain demographic, behavioral and medical information. Weight, height, and waist circumference were measured and body mass index (BMI) was calculated. Blood pressure was recorded using an electronic instrument (Model: HEM-7101, Omron Corporation, Tokyo, Japan) as the mean of two readings taken five minutes apart.

Fasting capillary blood glucose [CBG] was determined using One Touch Ultra glucose meter (Johnson & Johnson, Milpitas, California) after eight hours of overnight fasting. Oral glucose 82.5 grams [equivalent to 75 grams of anhydrous glucose] was given and a 2-hour post load CBG was collected. In individuals with self-reported diabetes, only fasting CBG was measured.

In addition, in every fifth subject (n = 2,042), a fasting venous sample was collected and lipids were measured. Samples were centrifuged within 1 hour at the survey site, and serum was transferred to separate labeled vials and temporarily stored in cold boxes until they were transferred to −80° freezers in the central laboratory of the Madras Diabetes Research Foundation at Chennai. All biochemical assays were carried out by the same team of laboratory technicians using the same method, throughout the study period. The samples were assayed for total cholesterol, triglycerides and HDL cholesterol. Serum cholesterol (cholesterol esterase oxidase-peroxidase-amidopyrine method), serum triglycerides (glycerol phosphate oxidase-peroxidase-amidopyrine method), and high-density lipoprotein cholesterol (direct method poly-ethylene-glycol-pretreated enzymes) were measured using the Beckman Coulter AU 2700/480 Autoanalyser (Beckman AU [Olympus], Ireland). The intra– and inter-assay coefficients of variants (CV) for the biochemical assays ranged from 3.1% to 7.6%.

In every fifth subject, a semi-quantitative food frequency questionnaire was administered to collect detailed information on dietary intake over the past year. Dietary fat and oil intake was assessed as the amount of fat/oil used during cooking and/or added at the table.

### Definitions

**Dyslipidemia**: National Cholesterol Education Programme (NCEP) guidelines [10] were used for definition of dyslipidemia as follows:

**Hypercholesterolemia –** serum cholesterol levels ≥200 mg/dl (≥5.2 mmol/l).

**Hypertriglyceridemia –** serum triglyceride levels ≥150 mg/dl (≥1.7 mmol/l).

**Low HDL cholesterol –** HDL cholesterol levels <40 mg/dl (<1.04 mmol/l) for men and <50 mg/dl (<1.3 mmol/l) for women.

**High LDL cholesterol –** LDL cholesterol levels ≥130 mg/dl (≥3.4 mmol/l) calculated using the Friedewald equation.

**High total cholesterol to HDL-C ratio**: This is defined as a total cholesterol to HDL-C ratio of ≥4.5.

**Isolated hypercholesterolemia**: Serum cholesterol ≥200 mg/dl and triglycerides <150 mg/dl; **Isolated hypertriglyceridemia**: Serum triglycerides ≥150 mg/dl and cholesterol <200 mg/dl; **Isolated low HDL-C**: HDL-C ≤40 mg/dl (male) and ≤50 mg/dl (female) without hypertriglyceridemia or hypercholesterolemia.

**Diabetes**: Individuals diagnosed by a physician and on antidiabetic medications (self-reported) and/or those who had fasting CBG ≥126 mg/dl (≥7 mmol/L) and/or 2-hr post-glucose CBG value ≥220 mg/dl (≥12.2 mmol/L) [11].

**Impaired fasting glucose [IFG]**: Fasting CBG ≥110 mg/dl (≥6.1 mmol/L) and <126 mg/dl (<7 mmol/L) and 2-hr post-glucose value <160 mg/dl (<8.9 mmol/L) [11].

**Impaired glucose tolerance [IGT]**: Two-hour post-glucose CBG ≥160 mg/dl (≥8.9 mmol/L) but <220 mg/dl (<12.2 mmol/L) and fasting value <126 mg/dl (<7 mmol/L) [11].

**Prediabetes**: Individuals with IFG or IGT or both.

**Dysglycemia**: Presence of diabetes and / or prediabetes.

**Hypertension**: Individuals diagnosed by a physician and on antihypertensive medications (self-reported) and/or those who had systolic blood pressure ≥140 mmHg and/or diastolic blood pressure ≥90 mmHg – Joint National Committee 7 (JNC7) Criteria [12].

**Obesity**: Generalized obesity was defined as BMI ≥25 kg/m^2^; overweight as BMI 23–25 kg/m^2^ and abdominal obesity was defined as waist ≥90 cm (males), ≥80 cm (females) using Asia-Pacific guidelines for south Asians [13].

**Coronary Artery Disease (CAD)**: CAD was diagnosed based on positive medical history (documented myocardial infarction (MI), angina pectoris and coronary artery bypass graft) and/or ischemic changes on a conventional 12-lead ECG which included ST-segment depression (Minnesota codes 1-1-1 to 1-1-7) or Q-wave changes (Minnesota codes 4–1 to 4–2) [14].

**Physical activity**: Physical activity was assessed using the Global Physical Activity Questionnaire (GPAQ) developed by the World Health Organization (WHO) [15]. To assess physical activity, domain-wise metabolic equivalents (MET) scores were calculated and individuals were categorized as sedentary, moderate, and vigorous. For this article, physical activity was dichotomously coded as sedentary and active (moderate or vigorous activity).

### Analysis

Statistical analyses were performed using SAS statistical package (version9.0; SAS Institute, Inc., Cary, NC). Estimates are expressed as mean±standard deviation or proportions. To compare continuous variables, *t* tests were used while chi square tests were used to test differences in proportions. Multivariable logistic regression was performed to determine the factors independently associated with cholesterol, triglyceride and HDL cholesterol abnormalities. P-value *<*0.05 was considered significant. For projections, Government of India population projections for 2011 for the respective states/UT based on 2001 Census of India were used.

## Results

Table 1 presents the general characteristics of the study subjects based on the presence/absence of dyslipidemia. In all the four regions, subjects with any lipid abnormality, had significantly higher BMI (p<0.001) and waist circumference (p<0.05) (except females in Jharkhand) compared to those with no lipid abnormality. Systolic and diastolic blood pressure were significantly higher in those with any lipid abnormality (p<0.05) only in the state of Maharashtra. Alcohol consumption was significantly higher in those without lipid abnormality in Tamilnadu and Jharkhand. Education, occupation, income levels, self-reported diabetes, coronary artery disease and family history of diabetes were not significantly different between the groups in all regions.

**Table 1:** General characteristics of the study subjects based on the presence of dyslipidemia in all the four regions studied.

Variables	Tamilnadu		Maharashtra		Jharkhand		Chandigarh	
	No lipid abnormality (n = 152)	Subjects with any lipid abnormality (n = 505)	No lipid abnormality (n = 109)	Subjects with any lipid abnormality (n = 364)	No lipid abnormality (n = 81)	Subjects with any lipid abnormality (n = 329)	No lipid abnormality (n = 86)	Subjects with any lipid abnormality (n = 416)
Age (years)	41.2±14.9	42.7± 14.1	40.9±15.3	41.9±13.8	40.0±14.3	37.5±13.0	33.7±11.2	36.2±12.1
Male n(%)	103 (67.8)	229 (45.3)**	63 (57.8)	179 (49.2)	58 (71.6)	163 (49.5)**	53 (61.6)	223 (53.6)
Body mass index (kg/m^2^)	20.5±3.6	22.7±4.3**	19.7±3.6	21.6±4.0**	19.0±2.3	21.0±4.2**	21.5±3.7	24.0±4.8**
Waist circumference (cm)								
Male	75.6±10.2	82.8±12.2**	75.2±10.9	79.8±10.4**	73.4±6.9	80.2±13.1*	77.6±10.7	85.7±12.5**
Female	69.9±11.1	75.8±11.4*	63.9±10.4	71.8±11.3**	68.6±9.7	72±11.7	73.5±10.4	80.3±13.4
Systolic blood pressure (mm Hg)	134±24	138±22	122±14	133±20*	132±16	132±17	125±10	128±18
Diastolic blood pressure (mm Hg)	78±15	83±13	73±7	81±10*	79±12	81±9	80±10	78±13
Serum cholesterol (mg/dl)	157±22	170±44*	155±22	162±39	136±25	138±38	151±23	162±41*
Serum triglycerides (mg/dl)	82	130**	82	118**	82	113**	89	142**
HDL cholesterol (mg/dl)	52±9	36±9**	54±13	37±11**	50±8	34±8**	51±10	36±9**
LDL cholesterol (mg/dl)	89±21	103±34**	84±23	96±34*	69±22	77±29*	81±22	92±33*
Total cholesterol: HDL ratio	3.1±0.6	5±2.7**	3.0±0.7	4.7±2.1**	2.8±0.5	4.2±1.4**	30.0±0.6	4.7±1.5**
Current smoking n(%)	13 (19.7)	83 (16.4)	14 (12.8)	27 (7.4)	11 (13.6)	28 (8.5)	17 (19.8)	71 (17.1)
Current alcohol users n(%)	40 (26.3)	82 (16.2)*	9 (8.3)	32 (8.8)	33 (40.7)	76 (23.1)*	16 (18.8)	54 (13.0)
Education level n(%)								
Illiterate	43 (28.3)	144 (28.5)	27 (24.8)	107 (29.4)	27 (33.3)	148 (45.0)	18 (20.9)	69 (16.6)
Primary, middle or high schooling	101 (66.4)	325 (64.4)	74 (67.9)	229 (62.9)	44 (54.3)	158 (48.0)	57 (66.3)	297 (71.4)
Graduate and above	8 (5.3)	36 (7.1)	8 (7.3)	28 (7.7)	10 (12.3)	23 (7.0)	11 (12.8)	50 (12.0)
Occupation n(%)								
Professional	4 (3.3)	22 (4.8)	3 (3.1)	13 (3.8)	10 (15.6)	15 (5.1)	5 (6.8)	19 (5.1)
Labourer	74 (61.7)	255 (55.2)	63 (64.9)	200 (58.7)	29 (45.3)	124 (42.2)	40 (54.1)	157 (41.9)
Others	42 (35.0)	185 (40.0)	31 (32.0)	128 (37.5)	25 (39.1)	155 (52.7)	29 (39.2)	199 (53.1)
Income level n(%)								
<5,000 INR	124 (83.2)	393 (79.4)	82 (75.2)	259 (72.8)	59 (77.6)	208 (77.0)	26 (31.7)	166 (43.5)
5,000–10,000 INR	14 (9.4)	76 (15.4)	15 (13.8)	65 (18.3)	13 (17.1)	27 (10.0)	33 (40.2)	105 (27.5)
>10,000 INR	11 (7.4)	26 (5.3)	12 (11.0)	32 (9.0)	4 (5.3)	35 (13.0)	23 (28.0)	111 (29.1)
Self reported diabetes n(%)	5 (3.3)	33 (6.5)	1 (0.9)	14 (3.8)	1 (1.2)	8 (2.4)	0	24 (5.8)
Coronary artery disease n(%)	5 (3.3)	29 (5.7)	3 (2.8)	17 (4.7)	2 (2.5)	9 (2.7)	0	10 (2.4)
Family history of diabetes n(%)	17 (11.2)	88 (17.4)	2 (1.8)	24 (6.6)	3 (3.7)	22 (6.7)	7 (8.1)	43 (10.3)

*p<0.05 and **p<0.001 compared to subjects with no lipid abnormality.

Table 2 presents the prevalence of lipid abnormalities in all the four regions studied. Overall, in the four regions studied, prevalence of atleast one lipid abnormality was 79%, with highest rates found in Chandigarh (82.9%), followed by Jharkhand (80%), Tamilnadu (76.9%) and Maharashtra (77%) with no urban rural differences observed in any of the four regions. Hypercholesterolemia was highest in Tamilnadu (18.3%) and least in Jharkhand (4.9%) and was significantly higher in urban compared to rural areas in all regions except Maharashtra. Hypertriglyceridemia was highest in Chandigarh (38.6%) and least in Maharashtra (22.8%) and the rates were significantly higher in urban compared to rural areas in Jharkhand and Chandigarh. Low HDL-C was highest in Jharkhand (76.8%) and least in Tamilnadu (68.9%) and no urban rural differences were observed in any of the four regions. High LDL-C was highest in Tamilnadu (15.8%), followed by Maharashtra (13.3%), Chandigarh (12%) and Jharkhand (3.4%), with high rates in urban areas compared to rural areas, except in Maharashtra. High cholesterol to HDL-C ratio was highest in Tamilnadu (42.3%), followed by Chandigarh (40.4%), Maharashtra (34.5%) and Jharkhand (25.4%) and the rates were significantly higher in urban compared to rural areas in all regions except in Maharashtra.

**Table 2:** Prevalence of dyslipidemia in all the four regions studied.

Prevalence	All	four	regions		Tamilnadu			Maharashtra			Jhar khand			Chandi garh	
	Urban	Rural	Overall	Urban	Rural	Overall	Urban	Rural	Overall	Urban	Rural	Overall	Urban	Rural	Overall
n	590	1452	2042	194	463	657	115	358	473	133	277	410	148	354	502
Hypercholesterolemia	19.2	11.7**	13.9	23.7	16.0*	18.3	13.9	13.7	13.7	10.5	2.2**	4.9	25.0	11.6**	15.5
Hypertriglyceridemia	36.6	26.6**	29.5	33.0	29.6	30.6	28.7	20.9	22.8	38.3	17.3**	24.1	45.9	35.6*	38.6
Low HDL cholesterol	73.2	71.9	72.3	72.2	67.6	68.9	69.6	69.6	69.6	74.4	78.0	76.8	76.4	75.1	75.5
High LDL cholesterol	15.9	10.1**	11.8	22.2	13.2*	15.8	12.2	13.7	13.3	7.5	1.4*	3.4	18.2	9.3*	12.0
High total cholesterol: HDL ratio	44.2	33.5**	36.6	48.5	39.7*	42.3	33.9	34.6	34.5	38.3	19.1**	25.4	52.0	35.6**	40.4
Isolated hypercholesterolemia	4.6	4.5	4.6	5.7	5.8	5.8	5.2	6.7	6.3	3.0	1.1	1.7	4.1	3.4	3.6
Isolated hypertriglyceridemia	22.0	19.4	20.2	14.9	19.4	18.1	20.0	14.0	15.4	30.8	16.2*	21.0	25.0	27.4	26.7
Isolated low HDL-C	39.0	47.2*	44.9	39.2	41.0	40.5	41.7	49.2	47.4	39.1	61.7**	54.4	36.5	42.1	40.4

*p<0.05 and **p<0.001 compared to urban participants; Dyslipidemia was diagnosed using NCEP guidelines – hypercholesterolemia: Total cholesterol (TC) ≥200 mg/dl; hypertriglyceridemia: triglycerides ≥150 mg/dl; low HDL cholesterol: HDL-C <40 (males) and <50 mg/dl (females); high LDL cholesterol: LDL-C ≥130 mg/dl; High total cholesterol: HDL-C ratio ≥4.5; Isolated hypercholesterolemia: TC ≥200 mg/dl and triglycerides <150 mg/dl; Isolated hypertriglyceridemia: serum triglycerides ≥150 mg/dl and TC <200 mg/dl; Isolated low HDL-C: HDL-C ≤40 mg/dl (male) and ≤50 mg/dl (female) without hypertriglyceridemia or hypercholesterolemia.

Isolated hypercholesterolemia was highest in Maharashtra (6.3%) and least in Jharkhand (1.7%), with no urban-rural differences in any region. Isolated hypertriglyceridemia was highest in Chandigarh (26.7%) and least in Maharashtra (15.4%), with urban-rural differences only in Jharkhand (urban: 30.8% vs. rural: 16.2, p<0.05). Isolated low HDL-C was strikingly high in Jharkhand (54.4%), with 39.1% in urban compared to 61.7% in rural Jharkhand (p<0.001).

**Figure 1:**
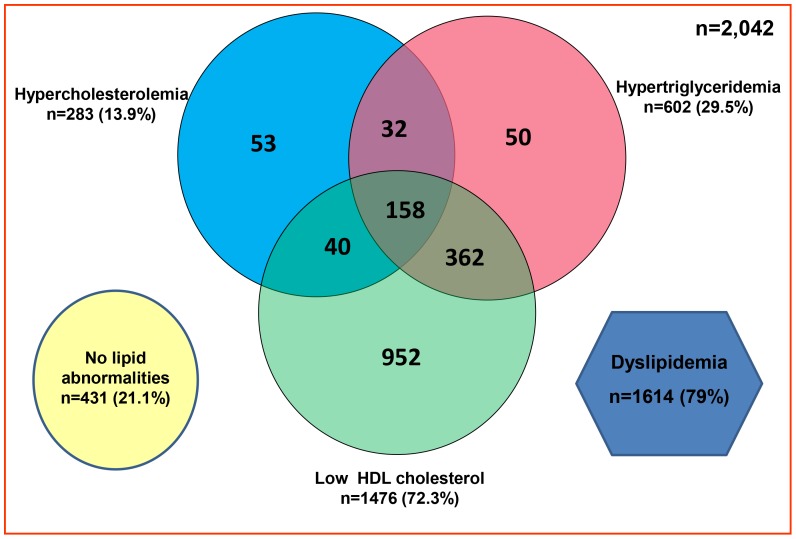
Venn diagram to show the overlap of the individual components of dyslipidemia [Hypercholesterolemia, hypertriglyceridemia and low HDL-cholesterol].

Figure 1 shows the overlap of the individual components of dyslipidemia. About 7.7% (n = 158) of the adult population had three lipid abnormalities (hypercholesterolemia + hypertriglyceridemia + low HDL-C) and 4.8% (n = 99) of the population had all four lipid abnormalities (hypercholesterolemia + hypertriglyceridemia + low HDL-C + high LDL-C). Only 21.1% (n = 431) had no lipid abnormality.

**Figure 2:**
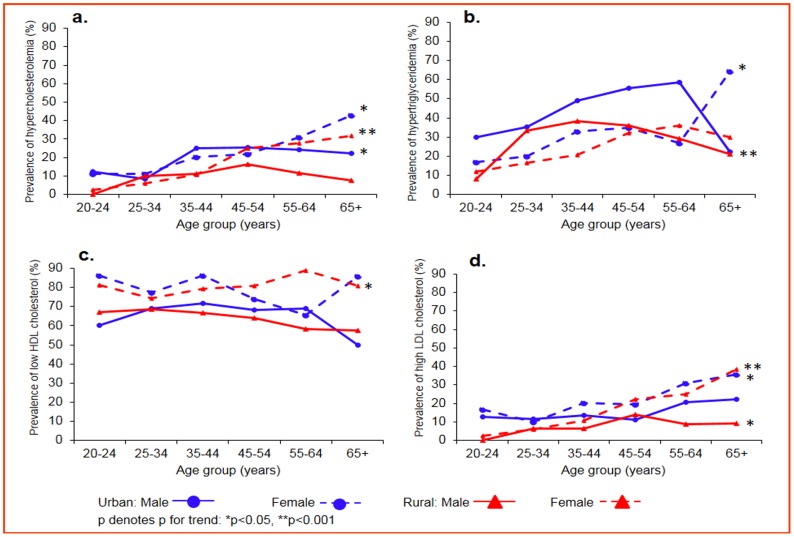
a-d: Age- and sex-specific prevalence of dyslipidemia in the study population. Urban male, blue circles, solid line; urban female, blue circles, dotted line; rural male, red triangles, solid line; rural female, red triangles, dotted line; p denotes p for trend; *p<0.05, **p<0.001.

Figures 2a-d presents the age- and sex-specific prevalence of all four lipid abnormalities. Overall, females had significantly higher rates of lipid abnormalities than males as did those in urban areas compared to rural areas, except for hypertriglyceridemia which was higher in males. An increasing trend with age was observed for hypercholesterolemia among urban males (p<0.05), urban females (p<0.05) and rural females (p<0.001); for hypertriglyceridemia among urban females (p<0.05) and rural females (p<0.001); for low HDL-C among rural females (p<0.05) and for high LDL-C among urban females (p<0.05), rural males (p<0.05) and rural females (p<0.001).

The mean values of lipids in all the four regions are shown in Table 3. Of the four regions, the mean cholesterol, LDL-C and cholesterol to HDL-C ratio were highest in Tamilnadu, triglycerides highest in Chandigarh and HDL-C lowest in Jharkhand. Statistically significant urban rural differences in mean lipid levels were observed in Tamilnadu (for cholesterol, triglycerides, HDL-C, LDL-C and cholesterol to HDL-C ratio), Jharkhand (for cholesterol, HDL-C, LDL-C and cholesterol to HDL-C ratio) and Chandigarh (for cholesterol, LDL-C and cholesterol to HDL-C ratio). No urban rural differences in any of the lipid levels were observed in Maharashtra.

**Table 3:** Mean values of lipids in all the four regions studied.

Variables	All	four	regions		Tamil nadu			Maharashtra			Jhar khand			Chandigarh	
	Urban	Rural	Overall	Urban	Rural	Overall	Urban	Rural	Overall	Urban	Rural	Overall	Urban	Rural	Overall
n	590	1452	2042	194	463	657	115	358	473	133	277	410	148	354	502
Serum cholesterol (mg/dl)	167±43	154±38**	158±40	173±48	165±37*	167±41	164±35	159±36	160±36	158±40	128±29**	138±36	171±41	156±37**	160±39
Serum triglycerides (mg/dl)	128	111**	116	123	114*	116	118	106	109	132	96**	106	141	127	131
HDL cholesterol (mg/dl)	39±12	39±12	39±12	38±10	40±12*	40±11	42±16	40±13	41±14	38±11	37±10**	37±10	39±11	39±11	39±11
LDL cholesterol (mg/dl)	97±33	89±32**	91±32	104±36	98±30*	100±32	94±29	94±33	94±32	88±31	70±23**	76±28	99±33	87±31**	90±32
Total cholesterol: HDL ratio	4.7±2.7	4.2±1.6**	4.3±2.0	5.0±3.9	4.4±1.5*	4.6±2.5	4.4±2.1	4.3±1.9	4.3±2.0	4.5±1.8	3.7±1.1**	4.0±1.4	4.6±1.6	4.3±1.5*	4.4±1.5

*p<0.05 and **p<0.001 compared to urban participants; Values are presented as mean ± SD, except for serum triglycerides, where the values are presented as geometric mean.

Multivariate logistic regression models were used to identify factors independently associated with lipid abnormalities in the study population [Table 4]. Hypercholesterolemia was strongly and positively associated with age≥60 years, urban residence, high income, overweight, generalized obesity, abdominal obesity, fat & oil intake (above median), diabetes, prediabetes and hypertension. Hypertriglyceridemia was positively associated with all factors entered in the model, except age≥60 years. Low HDL-C was positively associated with female gender, generalized obesity, abdominal obesity, sedentary lifestyle and diabetes. It was negatively associated with literacy, current tobacco smoking and alcohol. High LDL-C was positively associated with all factors entered in the model, except for literacy, current tobacco smoking and alcohol, which were negatively associated.

**Table 4:** Multivariable logistic regression showing factors independently associated with dyslipidemia in all the four regions studied.

Risk factors	All states pooled [Odds ratio (OR) [95% CI], p value]			
	Hypercholesterolemia	Hypertriglyceridemia	Low HDL cholesterol	High LDL cholesterol
Age ≥60 years	1.52 (1.08–2.14), p = 0.018	1.04 (0.78–1.39), p = 0.767	0.85 (0.64–1.13), p = 0.264	2.02 (1.43–2.86), p<0.001
Female	1.21 (0.94–1.55), p = 0.143	0.60 (0.49–0.72), p<0.001	2.00 (1.64–2.45), p<0.001	1.72 (1.31–2.25), p<0.001
Urban resident	1.79 (1.38–2.32), p<0.001	1.60 (1.30–1.96), p<0.001	1.07 (0.86–1.33), p = 0.546	1.68 (1.27–2.22), p<0.001
Literacy	1.31 (0.98–1.75), p = 0.070	1.27 (1.02–1.57), p = 0.033	0.79 (0.63–0.98), p = 0.032	1.30 (0.95–1.77), p = 0.102
Income ≥5000 INR	1.63 (1.25–2.13), p<0.001	1.48 (1.20–1.82), p<0.001	0.96 (0.78–1.19), p = 0.711	1.59 (1.20–2.12), p = 0.001
Overweight [BMI 23–25 kg/m^2^]	1.53 (1.08–2.17), p = 0.016	1.64 (1.25–2.16), p<0.001	1.26 (0.93–1.72), p = 0.142	1.68 (1.17–2.41), p = 0.005
Generalized obesity [BMI ≥25 kg/m^2^]	2.80 (2.15–3.66), p<0.001	3.49 (2.81–4.34), p<0.001	2.15 (1.65–2.80), p<0.001	2.73 (2.06–3.62), p<0.001
Abdominal obesity [Waist: ≥80 cm (m), ≥90 cm (f)]	3.41 (2.63–4.42), p<0.001	4.15 (3.36–5.12), p<0.001	2.29 (1.79–2.94), p<0.001	2.83 (2.15–3.74), p<0.001
Current smoker	1.26 (0.89–1.78), p = 0.190	2.08 (1.61–2.70), p<0.001	0.68 (0.52–0.88), p = 0.004	0.63 (0.40–0.99), p = 0.045
Alcohol consumer	1.20 (0.87–1.66), p = 0.260	1.57 (1.23–2.00), p<0.001	0.55 (0.43–0.71), p<0.001	0.60 (0.39–0.90), p = 0.015
Sedentary lifestyle	1.29 (0.99–1.70), p = 0.064	1.43 (1.17–1.76), p = 0.001	1.37 (1.11–1.68), p = 0.003	1.42 (1.05–1.90), p = 0.021
Fat & oil intake [above median: ≥31.5 g]	1.47 (1.09–1.97), p = 0.011	1.37 (1.10–1.17), p = 0.005	0.92 (0.74–1.14), p = 0.443	1.18 (1.09–2.03), p = 0.013
Diabetes [self reported and/or newly diagnosed]	2.53 (1.80–3.56), p<0.001	4.85 (3.58–6.56), p<0.001	2.60 (1.72–3.93), p<0.001	2.14 (1.48–3.09), p<0.001
Prediabetes [Impaired fasting glucose/impaired glucose tolerance]	1.83 (1.28–2.60), p = 0.001	1.77 (1.32–2.37), p<0.001	1.19 (0.86–1.65), p = 0.289	2.06 (1.43–2.96), p<0.001
Dysglycemia (diabetes/prediabetes)	2.47 (1.88–3.24), p<0.001	3.41 (2.73–4.26), p<0.001	1.78 (1.37–2.32), p<0.001	2.39 (1.79–3.20), p<0.001
Hypertension [self- reported and/or BP ≥140/90 mm Hg]	2.13 (1.64–2.76), p<0.001	2.45 (1.99–3.00), p<0.001	1.10 (0.89–1.37), p = 0.384	1.84 (1.39–2.43), p<0.001

Values are presented as Odds ratio OR (95% Confidence Interval, CI), p value.

## Discussion

The significant findings of the present study are: (1) Over three-fourth (79%) of the general adult population covered in this survey have abnormalities in at least one of the lipid parameters with no urban rural difference observed in any of the four regions (2) Hypercholesterolemia was found in 13.9%, hypertriglyceridemia in 29.5%, low HDL-C in 72.3% and high LDL-C in 11.8% of the population (3) Regional disparity exists in the prevalence rates with highest rates of hypercholesterolemia observed in Tamilnadu (18.3%), highest rates of hypertriglyceridemia in Chandigarh (38.6%), highest rates of low HDL-C in Jharkhand (76.8%) and highest rates of high LDL-C in Tamilnadu (15.8%) (4) Isolated HDL-C was strikingly high in Jharkhand and more so in rural Jharkhand (61.7%) compared to urban Jharkhand (39.1%) (5) Even the youngest age group (20–24 years) has high rates of dyslipidemia and over 80% of subjects in the age range of 35–64 years had abnormalities in one of the lipid parameters (6) Factors strongly associated with dyslipidemia included female gender, obesity, sedentary lifestyle, diabetes, dysglycemia and hypertension.

We report the presence of abnormalities in at least one lipid parameter as 76.9%, 77%, 80% and 82.9%, which translates to 35.9 million, 55.5 million, 14.5 million and 7.6 million individuals with dyslipidemia in Tamilnadu, Maharashtra, Jharkhand and Chandigarh respectively.

A study among Asian Indian immigrants in the United States (n = 1038), reported a prevalence of hypercholesterolemia of 43.5%, hypertriglyceridemia of 42.3%, low HDL-C of 26.4% and high LDL-C of 41.4% [16]. Similar rates were reported among Jordanian adults (n = 1121), with a prevalence of 48.8% of hypercholesterolemia, 43.6% of hypertriglyceridemia, 40.1% of low HDL-C and 40.7% of high LDL-C [17]. In a study conducted in Turkey [18], the authors reported that the prevalence of high TC, high triglycerides, low HDL-C and high LDL-C were 37.5%, 30.4%, 21.1% and 44.5% respectively. A study among rural adults in Pakistan (n = 1658), reported the prevalence of hypercholesterolemia, hypertriglyceridemia, low HDL-C and high LDL-C as 30.6%, 29.4%, 79.6% and 41.2% respectively [19]. The current study rates are lower than these studies, except for low HDL-C, owing to different lipid cut points or selection of different subgroups.

The ICMR surveillance project observed that the prevalence of dyslipidemia (ratio of total cholesterol to HDL cholesterol ≥4·5) was 37.5% in individuals aged 15–64 years [20]. In a study among adults aged 18 years and older (n = 200) in a resettlement colony located in central Delhi, 34% had increased total cholesterol levels, 40% had increased triglyceride levels, 42% had low HDL-C levels (<40 mg/dl) and 38% had high LDL-C (≥130 mg/dl) [21]. Gupta et al [22] reported a prevalence of 28% for hypercholesterolemia in the urban areas of Rajasthan, among adults aged 20 years and older. Misra et al [23] reported a prevalence of hypercholesterolemia (male: 26.8%, female: 27.5%), hypertriglyceridemia (male: 16.8%, female: 12.3%) low HDL-C (male: 15.8%, female: 16.7%) and high LDL-C as (male: 26%, female: 25.4%) among adults aged 25 years and older (n = 532), in the urban slums of northern India. However, this study used different cut points (hypertriglyceridemia >200 mg/dl and low HDL-C <35 mg/dl). Parikh et al [24] reported the prevalence of dyslipidemia (triglycerides ≥150 mg/dl or LDL-C ≥100 mg/dl or HDL-C <40 mg/dl for males and <50 mg/dl for females) among diabetic patients (n = 788) from clinic records as 85.5% in males and 97.8% in females. The prevalence of dyslipidemia (cholesterol ≥200 mg/dl or triglycerides ≥150 mg/dl or LDL-C ≥130 mg/dl or HDL-C <40 mg/dl for males and <50 mg/dl for females) was reported to be 40.6% among rural population of elderly (aged 60 years and above) in Wardha district, Central India [25]. These figures imply that though the prevalence of dyslipidemia varies from region to region, it is certainly quite high irrespective of the definitions and cut points used. Thus the increased risk of CVD in the Indian population could be partly due to the high prevalence of dyslipidemia.

Despite the regional differences, low HDL-C was the most common lipid abnormality in all the four regions studied and 44.9% had isolated low HDL cholesterol. This study thus confirms the findings of several earlier studies that Indians have high prevalence of low HDL cholesterol. This appears to be part of the Asian Indian phenotype [26] which includes increased plasma insulin levels, insulin resistance, increased waist circumference, excess visceral fat and low adiponectin levels.

In the present study, the mean cholesterol levels in urban subjects were higher than their rural counterparts, whereas no urban rural differences were observed in terms of triglycerides and HDL-C. Similar to our findings, most surveys have also shown higher mean concentrations of cholesterol in urban subjects (178–201 mg/dl) compared with rural subjects (166–178 mg/dl), with a low mean concentration of HDL cholesterol [27]. While these levels were lower than those seen in other ethnic populations, studies have shown that South Asians manifest CVD at lower levels of total cholesterol compared with other ethnic groups [28].

The present study shows that obesity, dysglycemia and hypertension were strongly associated with dyslipidemia. This link has been well proved and the tendency for certain CVD risk factors to cluster, such as obesity, insulin resistance, glucose intolerance, dyslipidemia and hypertension, has been recognized for many years and termed as the metabolic syndrome [29].

Our study is limited by its cross-sectional nature; therefore, causal pathways underlying the reported relationships cannot be inferred. Information on lipids were available only on every fifth subject, as this paper presents sub analyses of work done as part of a larger study on diabetes in India and this is one of the limitations of the study. Information on lipid-lowering therapy was not collected in this study and analyses on lipoproteins and genetic polymorphisms were not performed as they are difficult to do in large epidemiological studies and this is another limitation of the study. However, they can be considered in future studies as such data would be very valuable. The strengths of the study are that it is population-based, large, and representative of the general population with a good response rate.

Worldwide, cardiovascular disease is estimated to be the leading cause of death and loss of disability-adjusted life years [30]. Although age-adjusted cardiovascular death rates have declined in several developed countries in past decades, rates of cardiovascular disease have risen greatly in low-income and middle-income countries [30], with about 80% of the burden now occurring in these countries. In view of this, all efforts need to be taken to clearly understand the role of risk factors in the emerging epidemic, for its effective control. Presence of dyslipidemia even among the young adults as observed in this study is distressing and thus screening right from younger ages may help promote lifestyle changes that can prevent or slow atherogenesis. More importantly, a healthy lifestyle should be inculcated right from childhood stage to prevent this epidemic. Several randomized controlled trials have shown that effective treatment of dyslipidemia reduces the rate of morbidity and mortality [31]. The observations from the present study provide insight into the magnitude of the burden of dyslipidemia in India. This provides essential information to healthcare providers for resource allocation in a country with a large proportion of diabetic subjects [9] and an increased burden of CVD.
